# Complete mitochondrial DNA sequence of the paper wasp *Polistes riparius* (Hymenoptera: Vespidae)

**DOI:** 10.1080/23802359.2020.1810155

**Published:** 2020-08-25

**Authors:** Kazuhisa Yamasaki, Katsuhiko Sayama, Tomoki Oishi, Kanae Nakahama, Masato Yoshioka, Hisashi Okuyama, Jun-ichi Takahashi

**Affiliations:** a Faculty of Applied Biological Sciences, Gifu University, Gifu, Japan; b Institute for Sustainable Agro-ecosystem Services, Graduate School of Agricultural and Life Sciences, The University of Tokyo, Nishitokyo, Japan; c Hokkaido Research Center, Forestry and Forest Products Research Institute, Hitsujigaoka, Japan; d Faculty of Life Sciences, Kyoto Sangyo University, Kyoto, Japan

**Keywords:** Next generation sequence, paper wasp, *Polistes riparius*, Polistinae, Vespidae

## Abstract

The paper wasp *Polistes riparius* is distributed in cold regions of northern East Asia to Russia. *P. riparius* are characterized by having longer cells than those of the closely related *P. chinensis*, which has a similar life history, as an adaptation to cold regions. The phylogenetic relationships of paper wasps have recently been studied; however, the genetic diversity and population structure of *P. riparius* has not been determined. The present study is the first to analyze the complete mitochondrial genome using next generation sequencing of *P. riparius* collected from Sapporo, Hokkaido Prefecture, Japan. The genome consisted of a closed loop that was 16,383 bp-long and included 13 protein coding genes (PCGs), 22 tRNA genes, two rRNA genes, and one AT-rich control region. The average AT content was 84.54%. The heavy (H)-strand was predicted to have 12 PCGs and 14 tRNA genes, while the light (L)-strand was predicted to contain one PCGs, eight tRNA genes, and two rRNA genes. All PCGs started with ATG. Stop codons were of two types: TAA for 11 genes (*ND1, ND2, ND3, ND4L, ND5, ND6, COXI, COXII, COXIII, COB, ATP6* and *ATP8*) and TAG for two genes (*ND3* and *ND4*). The molecular phylogenetic relationship based on the maximum likelihood method using 13 PCGs was consistent with some previous studies in which a closely relationship between *P. riparius* and *P. jokahamae*.

*Polistes* is a species-rich genus of paper wasps that includes more than 200 species (Carpenter 1996). They have long been treated as model materials for studying cooperation, competitive breeding, and social evolution in Hymenoptera because they are relatively nonaggressive and have small observable colonies with exposed nests. *Polistes riparius* is distributed in the cold regions of northern East Asia to Russia (Yamane and Yamane 1987; Kurzenko 1995; Dubatolov 1998). Although *P. riparius* has a life history that is similar to the closely related *P. chinensis*, it is characterized by developing longer cells than those of *P. chinensis*, possibly as a heat-retaining mechanism (Yamane and Kawamichi 1975; Yamane et al. 1998). The phylogenetic relationships of paper wasps were recently studied based on the analysis of partial sequences of mitochondrial DNA (Santos et al. 2015). Currently the genetic diversity and population structure of this species, and its characteristic ecological adaptations to cold regions, have not been identified. Our study analyzed the complete mitochondrial genome of *P. riparius*.

We collected adult *P*. *riparius* workers on flowers in Sapporo, Hokkaido Prefecture, Japan, in 2013 (42°57′N, 141°15′E). Adult workers were transferred immediately to 99.5% ethanol for mitochondrial DNA analysis. The specimens were stored in a freezer at −20 °C in our laboratory at the Kyoto Sangyo University. Genomic DNA isolated from one worker was sequenced using the Illumina MiSeq platform (Illumina, San Diego, CA, USA). The complete mitochondrial genome of the paper wasp *P. jokahamae* was used as a reference sequence. The resultant reads were assembled and annotated using the Geneious R9 software (Kearse et al. 2012) and the MITOS web server (Bernt et al. 2013). Thirteen protein-coding genes (PCGs) and two rRNA genes sequences were aligned using Genetyx version 15 (GENEYTX, Japan). The phylogenetic analysis was based on the nucleotide sequences of 13 protein-coding genes using MEGAX (Kumar et al. 2018).

We succeeded in sequencing the entire mitochondrial genome of *P*. *riparius* from Hokkaido Prefecture (DDBJ accession number LC519884). These specimens were stored as stocks at the National Museum of Nature and Science, Japan (control number NSMT-I-Hym75321). The genome consisted of a closed loop 16,383 bp long and included 13 PCGs, 22 tRNA genes, two rRNA genes, and one AT-rich control region that represented a typical Vespidae mitochondrial genome. The average AT content was 84.54%. The heavy strand was predicted to have 12 PCGs and 14 tRNA, while the light strand was predicted to contain one PCG, eight tRNA genes, and two rRNA genes. The start codon was ATG for the 13 PCGs. The stop codon was TAA for 11 PCGs, and TAG for two genes. The genes *ATP8* and *ATP6* shared 10 nucleotides, and *ATP6* and *COIII* shared one nucleotide. The genes *ND4* and *ND4L*, and genes *ND5* and *ND6* shared 7 and 4 nucleotides, respectively. A molecular phylogenetic analysis of 9 closely related taxa of Vespidae suggested sister relationships within the *Polistes* (Figure 1). The complete sequence of the mitochondrial DNA of *P. riparius* provides additional genetic information for studying the phylogenetic relationship of the genus *Polistes*.

**Figure 1.**
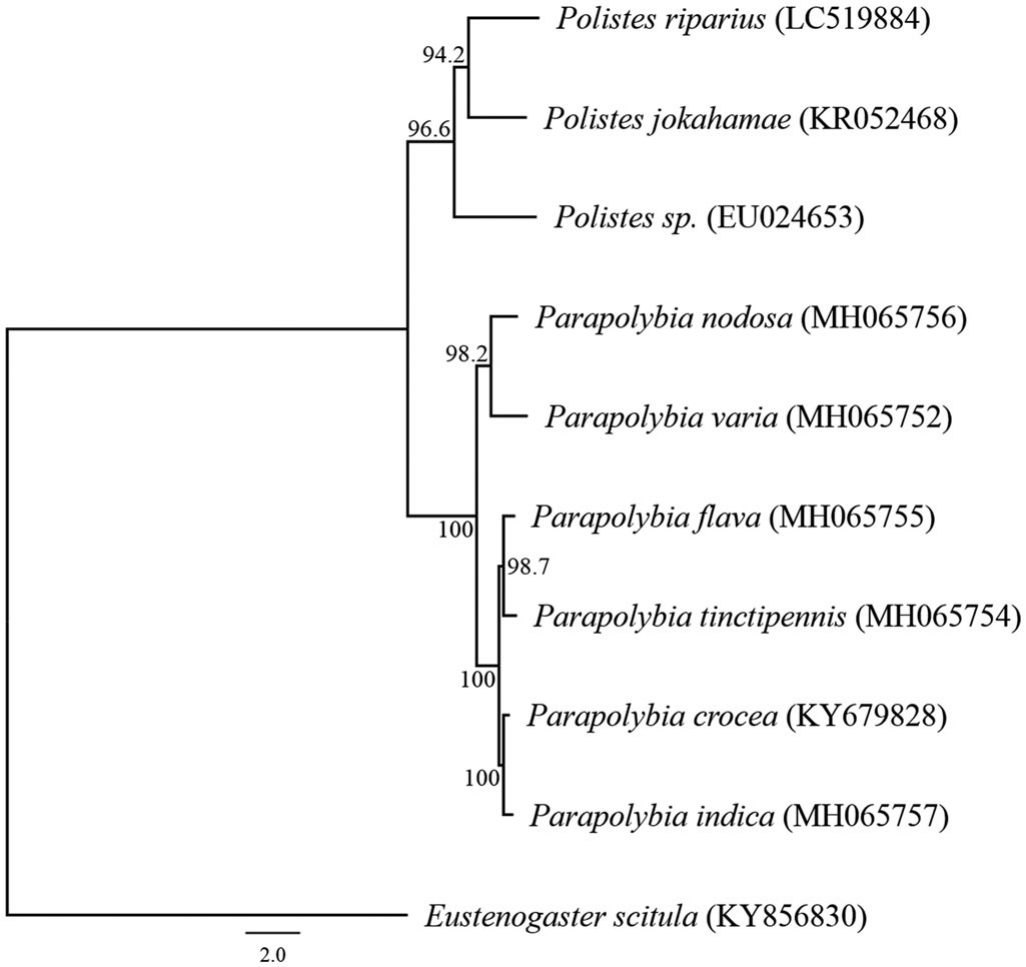
Phylogenetic relationships (maximum likelihood) of the Vespidae based on the nucleotide sequences of the 13 protein-coding genes of the mitochondrial genome. Sequences from *Eustenogaster scitula* (KY856830) was used as an outgroup. These sequences were separated by codon positions, and for each partition, the optimal models of sequence evolution were used in the maximum likelihood method using MEGA X, based on the corrected Akaike information criterion. The numbers at the nodes indicate the bootstrap support inferred from 1000 bootstrap replicates. Alphanumeric terms indicate the DNA Database of Japan accession numbers.

## Data Availability

The data that support the findings of this study are openly available in DDBJ/GenBank at https://www.ddbj.nig.ac.jp/index.html, accession number LC519884.
